# The Analysis of Multiple Outcomes between General and Regional Anesthesia in Hip Fracture Surgery: A Systematic Review and Meta-Analysis of Randomized Controlled Trials

**DOI:** 10.3390/jcm12247513

**Published:** 2023-12-05

**Authors:** Dmitriy Viderman, Mina Aubakirova, Fatima Nabidollayeva, Yerkin G. Abdildin

**Affiliations:** 1 Department of Surgery, School of Medicine, Nazarbayev University, Astana 020000, Kazakhstan; mina.aubakirova@nu.edu.kz; 2 School of Engineering and Digital Sciences, Nazarbayev University, Astana 010000, Kazakhstan; fatima.nabidollayeva@nu.edu.kz (F.N.); yerkin.abdildin@nu.edu.kz (Y.G.A.)

**Keywords:** general anesthesia, regional anesthesia, spinal anesthesia, epidural anesthesia, hip fracture, surgery, outcomes

## Abstract

Surgical interventions in hip fracture have been associated with multiple adverse events, including perioperative hypotension and mortality, making the choice of the anesthetic method for this procedure crucial. There is still no consensus on whether regional (RA) or general (GA) anesthesia should be used to maintain hemodynamic stability and more favorable outcomes. Therefore, this meta-analysis examines the differences between RA and GA groups in the incidence of mortality, intraoperative hypotension, and other intra- and postoperative complications. The comparison is essential given the rising global prevalence of hip fractures and the need to optimize anesthesia strategies for improved patient outcomes, particularly in an aging population. We followed PRISMA guidelines (PROSPERO #CRD42022320413). We conducted the search for studies published in English before March 2022 in PubMed, Google Scholar, and the Cochrane Library. We included RCTs that compared general and regional anesthesia in adult patients having hip fracture surgical interventions. The primary outcome was perioperative mortality. The secondary outcomes were peri- or postoperative complications and duration of hospital stay. We conducted a meta-analysis in RevMan (version 5.4). We examined the quality of the methodology with the Cochrane risk of bias 2 tool, while the quality of evidence was determined with GRADE. Fifteen studies with 4110 patients were included. Our findings revealed no significant difference between general and regional anesthesia in risk of perioperative mortality (RR = 1.42 [0.96, 2.10], *p*-value = 0.08), intraoperative complications, or duration of hospital length of stay. Our results suggest that regional anesthesia and general anesthesia have comparable safety and can be used as alternatives based on specific patient requirements.

## 1. Introduction

The number of new cases of hip fracture is projected to exceed two and a half million worldwide by the first quarter of the 21st century [1]. Hip fracture is associated with a substantial perioperative complication rate of 6–19% overall [2,3] and a mortality rate of 3–8% [4,5,6,7]. Among complications, hypotension poses a particular concern, especially in the frail elderly population, given its association with elevated mortality at 30 days [8,9]. Different methods of anesthetic techniques, fluid therapy, and vasopressors are used to maintain the stability of the mean arterial pressure (MAP) [10,11]. Moreover, directing fluid and vasopressor administration based on a thorough hemodynamic evaluation, conducted through preoperative echocardiography and noninvasive monitoring, is crucial due to the potential adverse events associated with hypo- and hypervolemia. Hypovolemia may lead to decreased preload, resulting in cardiac output reduction and inadequate organ perfusion, while hypervolemia can cause systemic and pulmonary congestion, leading to decreased organ function. Currently, there are several approaches to monitoring fluid responsiveness. One option is the visualization of the inferior vena cava (IVC) diameter with echocardiography from the subcostal region or using a coronal trans-hepatic approach, although more research is needed to determine the appropriate thresholds for fluid responsiveness when employing the latter [12]. Alternatively, continuous noninvasive blood pressure monitoring can be used as it has been associated with a lower incidence of hypotension and hypertension during general anesthesia compared to intermittent cuff measurement [13]. Furthermore, the use of artificial intelligence for continuous noninvasive monitoring of blood pressure during general anesthesia has demonstrated promising results in hemodynamic assessment [14]. Despite the various attempts to prevent hypotensive events and other adverse outcomes, a definitive agreement on the best anesthesia approach for this surgery has not been reached.

General anesthesia (GA) is still widely used in hip fracture surgery, yet multiple regional anesthetic (RA) techniques are also gaining popularity. Thus, spinal anesthesia (SA) is often favored over general anesthesia in patients with a higher susceptibility to complications due to its effectiveness, simplicity, and minimal impact on cognitive and pulmonary function [15]. In fact, between 2007 and 2017, the usage of SA for hip fracture surgery increased by 50% [16]. However, SA is associated with severe hypotension, as it reduces the body’s ability to compensate for changes in blood pressure, particularly in frail populations with numerous underlying health conditions [17]. On the other hand, continuous spinal anesthesia (CSA) has been shown to more effectively maintain hemodynamic stability compared with single-shot SA or GA thanks to low-fractionated administration of local anesthetic [10,11,18]. A recent meta-analysis also demonstrated that a 6.5 mg dose of SA was effective and associated with a lower incidence of hypotension compared to a 10.5 mg dose [19]. The authors suggest that a smaller dose provides an effective sensory block in conjunction with opiates through synergistic action of the two while minimizing systemic effects, including hemodynamic outcomes [19]. Similarly, multiple nerve blocks (MNBs) have been used as a GA alternative to minimize hypotensive episodes with some studies reporting promising results [20,21].

This systematic review with meta-analysis (SR&MA) aims to answer two main questions: Are there differences in death rates between the general and regional (SA, CSA, MNB) anesthesia groups? Are there differences in hypotension and other intraoperative and postoperative complications between the two groups?

## 2. Materials and Methods

### 2.1. Protocol

**Figure 1:**
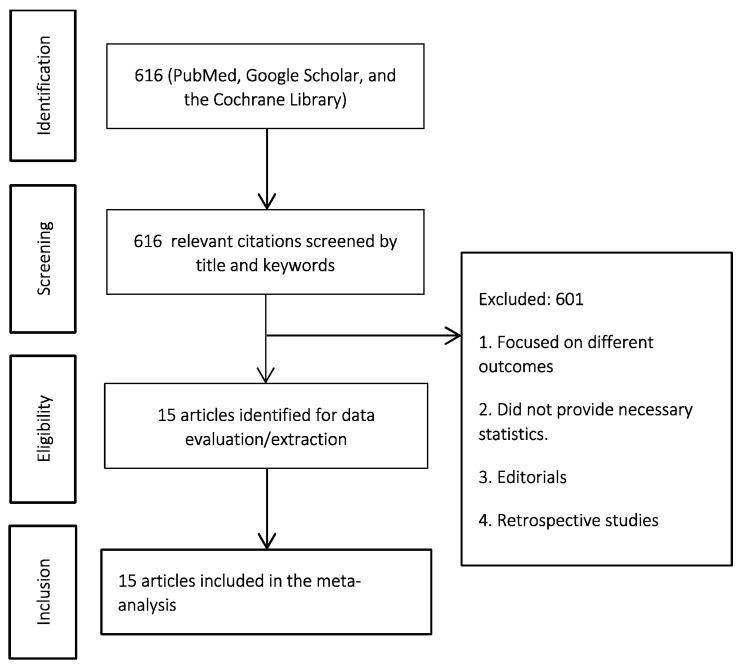
PRISMA diagram.

We conducted this study using the PRISMA guidelines [22]. PRISMA diagram is available in Figure 1. The protocol was developed prior to conducting the study and is publicly available in PROSPERO (#CRD42022320413). There were no deviations from the protocol.

### 2.2. Search Strategy and Criteria

The systematic search for relevant articles published before 15 March 2022 was performed using the following databases: PubMed, Google Scholar, and the Cochrane Library. The search terms used are available in Supplementary Materials. After searching the databases, a manual search was conducted by going through the references of the identified studies.

### 2.3. Screening

Screening of the articles was conducted by two authors in an independent manner. In case of disagreements, a third author was consulted. The studies were screened based on titles, then abstracts, and finally, by full texts. We included studies based on these criteria:

Inclusion criteria:

- Randomized controlled trials (RCTs);
- Adult patients with hip fractures undergoing surgical procedures;
- Comparing regional anesthesia versus general anesthesia;
- Reporting outcomes of interest: mortality (primary) and intra- and postoperative complications (secondary).

Exclusion criteria:

- Study designs other than RCTs;
- Pediatric studies;
- Not comparing regional to general anesthesia;
- Not reporting outcomes of interest.

Studies that did not meet the specified inclusion criteria were excluded.

### 2.4. Data Extraction and Statistical Methods

Two authors extracted data independently. Any disagreements were solved by consulting a third author. We extracted study characteristics (country, primary/secondary outcomes, sample size, age) in a data table (Supplementary Table S1). Numeric data on the outcomes of interest were extracted into a spreadsheet for further analysis. If a study did not report data on an outcome of interest for this meta-analysis, we did not include that study in the analysis of that outcome. The primary outcome was death, while the secondary ones were other adverse events and duration of hospitalization. For each outcome, the risk ratio or standardized mean difference was calculated, and sensitivity analysis was conducted. If required, we employed mathematical techniques to calculate the sample mean and standard deviation [23,24]. Given the differences in study populations and procedures, a high level of heterogeneity among the studies was anticipated. Therefore, the random effects model was employed for the analysis. A significance level of *p* < 0.05 was adopted. Forest plots were constructed for each outcome. To assess statistical heterogeneity, we utilized the I^2^ statistic. The data analysis was performed in the software “Review Manager (RevMan) [Computer program]. Version 5.4 (The Cochrane Collaboration, 2020, Copenhagen, Denmark)”.

### 2.5. Quality Assessment

The quality of the methodology of the studies included in the review was evaluated with the Cochrane Risk of Bias Tool 2 [25]. We evaluated each study as “low risk”, “some concerns”, or “high risk” of bias based on the “randomization process”, “deviations from the intended intervention”, “missing outcome data”, “measurement of the outcome”, and “selection of the reported results”. To assess the quality of the evidence of the main outcomes, we analyzed them with the GRADE [26]. We analyzed each outcome for “risk of bias”, “inconsistency”, “indirectness”, and “imprecision” and summarized the overall quality of the outcome as “high”, “moderate”, “low”, or “very low”.

## 3. Results

### 3.1. Included Studies

The systematic search yielded 616 articles. After duplicate removal and title screening, 15 RCTs comprising 4110 patients were identified for inclusion in the MA [27,28,29,30,31,32,33,34,35,36,37,38,39,40,41] (Figure 1, Table 1).

### 3.2. Mortality

**Figure 2:**
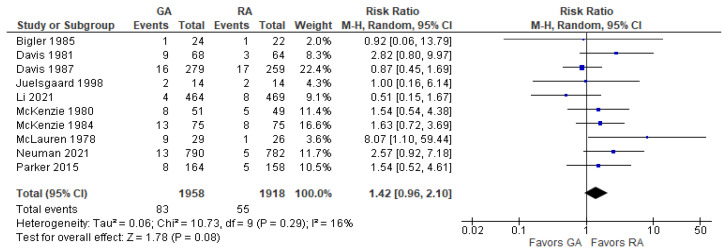
Death. Square—risk ratio for individual studies; line—confidence interval; diamond—pooled risk ratio [27,28,29,30,31,32,33,37,38].

There was no difference in the risk of death in the RA group compared to the GA group (RR = 1.42; 95% CI: [0.96, 2.10], *p*-value = 0.08) (Figure 2). Sensitivity analysis revealed that excluding either Davis et al. (1987) [29] or Li et al. (2022) [31] changed the result favoring RA. We should note that most included studies reported values for the period of four weeks or one month, Neuman et al. (2021) [37] reported values for the period of “after 60 days”, and Bigler et al. (1985) [27] did not mention the specific postoperative period.

### 3.3. Intraoperative Hypotension

**Figure 3:**
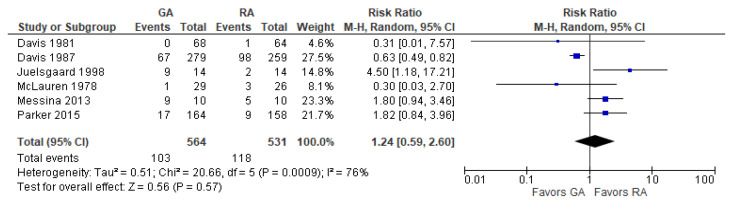
Intraoperative hypotension. Square—risk ratio for individual studies; line—confidence interval; diamond—pooled risk ratio [28,29,30,35,36,38].

We did not observe a difference between the RA and the GA groups in the risk of hypotension (RR = 1.24 [0.59, 2.60], *p* = 0.57) (Figure 3). Among the six studies with 1095 patients, there was substantial heterogeneity at I^2^ = 76%.

### 3.4. Cardiac and Cerebrovascular Complications

**Figure 4:**
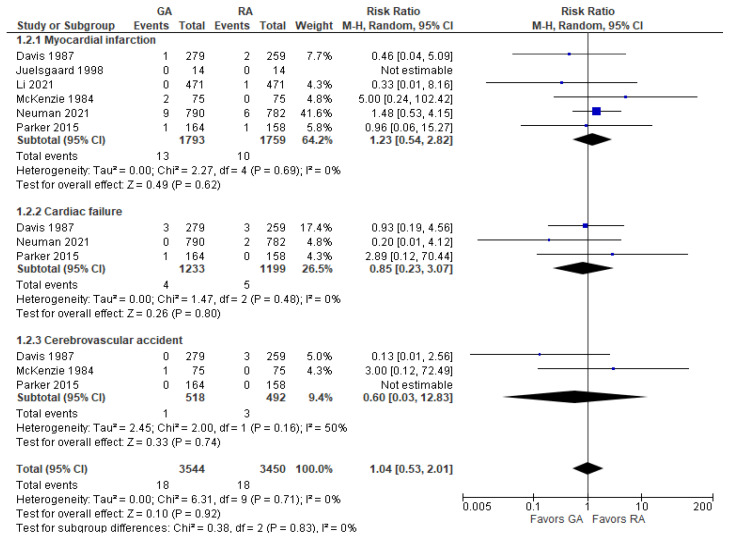
Cardiac and cerebrovascular complications. Square—risk ratio for individual studies; line—confidence interval; diamond—pooled risk ratio [29,30,31,33,37,38].

We combined myocardial infarction, cardiac failure, and cardiovascular accident into the overall cardiac and cerebrovascular complications outcome. There was no significant difference between the GA and RA groups in terms of myocardial infarction (RR = 1.23 [0.54, 2.82]), cardiac failure (RR = 0.85 [0.23, 3.07]), or cerebrovascular accident (RR = 0.60 [0.03, 12.83]). The lack of difference was maintained at the exclusion of any study (Figure 4).

### 3.5. Vascular Complications

**Figure 5:**
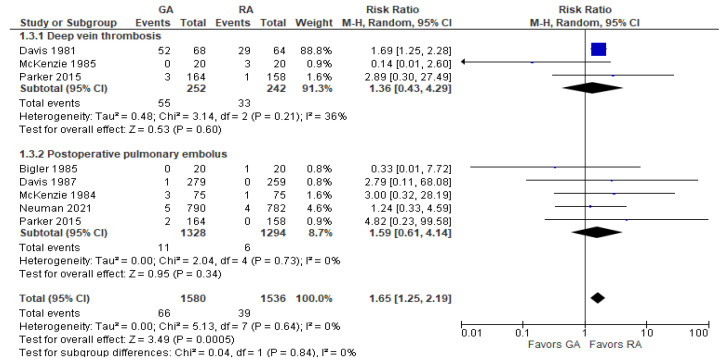
Vascular complications. Square—risk ratio for individual studies; line—confidence interval; diamond—pooled risk ratio [27,28,29,33,34,37,38].

For deep vein thrombosis, there was no difference between the two groups at RR = 1.36 [0.43, 4.29]. It should be mentioned that the result changed in favor of RA when the study by McKenzie et al. (1985) [34] was excluded. For postoperative pulmonary embolus, the results for the two groups were comparable at RR = 1.59 [0.61, 4.14] (Figure 5). The overall result of the model for vascular complications is in favor of RA.

### 3.6. Acute Kidney Disease

**Figure 6:**
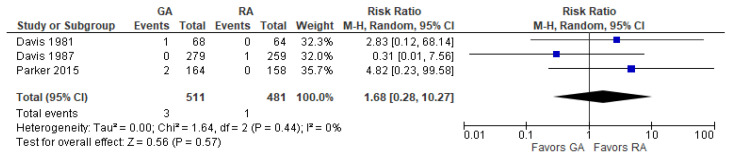
Acute kidney disease. Square—risk ratio for individual studies; line—confidence interval; diamond—pooled risk ratio [28,29,38].

The model does not favor RA over GA (Figure 6) since RR with 95% CI is equal to 1.68 [0.28, 10.27].

### 3.7. Postoperative Pneumonia

**Figure 7:**
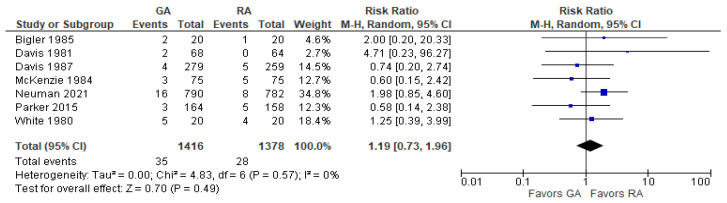
Postoperative pneumonia. Square—risk ratio for individual studies; line—confidence interval; diamond—pooled risk ratio [27,28,29,33,37,38,41].

The model does not favor RA over GA (Figure 7) since RR with 95% CI is equal to 1.19 [0.73, 1.96].

### 3.8. Intraoperative Blood Loss (mL)

**Figure 8:**
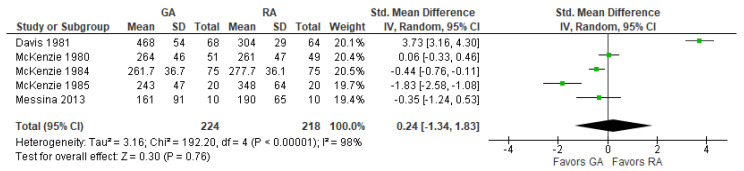
Intraoperative blood loss (mL). Square—risk ratio for individual studies; line—confidence interval; diamond—pooled risk ratio [28,32,33,34,36].

The model does not favor RA over GA (Figure 8) since the std. mean difference (SMD) with 95% CI is equal to 0.24 [−1.34, 1.83].

### 3.9. Perioperative Blood Transfusion

**Figure 9:**
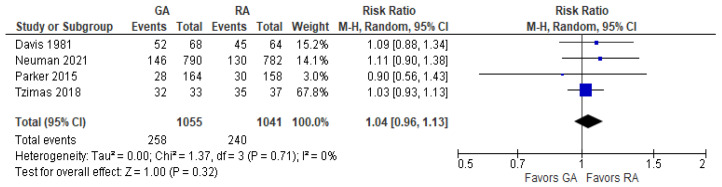
Perioperative blood transfusion. Square—risk ratio for individual studies; line—confidence interval; diamond—pooled risk ratio [27,37,38,40].

The model (Figure 9) does not favor RA over GA since RR with 95% CI is equal to 1.04 [0.96, 1.13].

### 3.10. Duration of Hospital Stay (Days)

**Figure 10:**
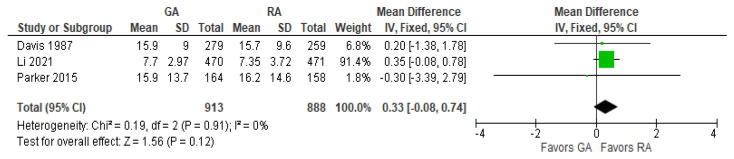
Duration of hospital stay (days). Green square—risk ratio for individual studies; line—confidence interval; diamond—pooled risk ratio [29,31,38].

The model does not favor RA over GA (Figure 10) since SMD with 95% CI is equal to 0.33 [−0.08, 0.74].

### 3.11. Quality Assessment

We report the Cochrane Risk of Bias 2 in Table 2. Given the nature of the intervention, group assignment could not be concealed from the patients, which contributed to the “risk of bias”. Moreover, the randomization process and concealment technique were not described in the older publications. However, these were published in reputable journals. Therefore, all the studies were rated as having “some concerns” in terms of risk of bias.

The results of the GRADE assessment of the main outcomes are presented in Table 3. The outcomes ranged in the quality of evidence from “low” to “very low” due to “risk of bias” (lack of blinding, lack of information concerning allocation concealment, etc.), “inconsistency” (unexplained heterogeneity and wide variance of point estimates), and “imprecision” (wide confidence intervals). The full description of the assessment is available in the Evidence profile (Table S1).

## 4. Discussion

There are controversies as to the most appropriate anesthetic approach in hip fracture surgeries to minimize the risk of complications, especially among the frail population. In this meta-analysis, we failed to identify the benefits of RA or GA for hip fracture surgery concerning mortality as well as intra- and postoperative complications.

The primary outcome was death. Although there was a trend toward decreased risk of mortality in the RA group at RR = 1.42 [0.96, 2.10], *p*-value = 0.08, we failed to reach statistical significance. Therefore, we observed no difference between the groups. On the contrary, previous observational studies concluded that GA might have an association with reduced incidence of mortality, adverse events, delirium, and shorter length of hospital stay compared with SA [42,43,44,45,46].

The secondary outcomes were intra- and postoperative complications. The results between the two groups were comparable. This finding is in agreement with a recent study that found no difference between the CSA/MNB and GA groups concerning postoperative complications and mortality rates in elderly patients undergoing hip fracture surgery [47]. However, in their study, CSA and MNB offered superior intraoperative blood pressure (BP) control than GA and comparable BP control between the regional anesthesia groups. Moreover, the MNB and CSA groups had a decreased frequency of cases of hypotension below 50 mmHg and requirement in vasopressors compared with the GA group.

One of the reasons for discrepancies in our results with previous literature might be the variations in the characteristics of the patient populations across studies, such as differences in age distribution, baseline health conditions, or comorbidities. These factors may interact differently with the chosen anesthesia methods, influencing mortality and other intra- and postoperative outcomes. Additionally, variations in surgical and anesthetic protocols, including drug dosages, administration techniques, and perioperative care, could contribute to differing results. Methodological dissimilarities, such as study design and blinding procedures, might also play a role in the observed differences. The evolution of medical practices over the study period, spanning three decades, could introduce disparities in outcomes due to advancements in surgical and anesthetic techniques.

Thus, our results suggest that the rate of death and adverse events in patients undergoing surgical procedures for hip fracture did not differ significantly between GA and RA, suggesting comparable safety of the two approaches. This might suggest that either approach can be used as an alternative based on specific patient requirements. For example, RA may be favored in patients with cardiovascular or pulmonary comorbidities, as it can offer better hemodynamic stability. RA was hypothesized to have minimal impact on cognitive function, making it a preferred option for elderly patients. However, a recent meta-analysis did not support this hypothesis [48]. Certain regional techniques, like Continuous Spinal Anesthesia or Multiple Nerve Blocks, may provide superior intraoperative blood pressure control compared to GA. On the other hand, patients with contraindications to regional techniques, such as severe coagulopathy or hemodynamic instability, may be more suitable for general anesthesia. GA might also be preferred in emergency cases or when a rapid onset of anesthesia is crucial. A recent study also proposed that the decision on the anesthesia type for hip fracture surgery may be influenced more by patient preference rather than solely relying on existing evidence and variations in clinical results [46]. For instance, some patients may prefer GA due to a desire for complete unconsciousness during the procedure. Ultimately, the choice between GA and RA should be made on a case-by-case basis, taking into account the patient’s medical history, preferences, and the specific clinical context. Shared decision-making between the patient and the healthcare team is crucial to ensure the most appropriate and individualized anesthesia approach for hip fracture surgery.

Thus, the comparable efficacy of GA and RA in hip fracture surgery has substantial implications for clinical decision-making. This finding supports a personalized approach to anesthesia selection, enabling clinicians to consider individual patient characteristics, such as cardiovascular and pulmonary comorbidities or the risk of postoperative cognitive dysfunction. Moreover, the study suggests considering patient preferences in anesthesia choice, promoting shared decision-making processes. Additionally, the study’s results suggest that in cases with contraindications to spinal anesthesia, GA remains a viable and safe option. Overall, the study’s insights enhance the practical relevance of clinical decision-making by providing a nuanced understanding of when and how to apply GA and RA based on individual patient needs and preferences.

There are several limitations in the body of evidence. The majority of the included studies were conducted over three decades ago, potentially misaligning with current standards in surgical and anesthetic practices. Moreover, the studies demonstrated significant methodological limitations, including a lack of blinding and an inadequate description of the randomization method, which may introduce bias. The review process itself had limitations, as it combined studies with variations in anesthetic regimens and blocks, and there were relative differences in patients’ conditions at admission, with some studies defining outcomes differently. Additionally, the inclusion of studies spanning over 40 years may have introduced variations in the quality and safety of surgical and anesthetic techniques. These limitations suggest that generalizing the findings to contemporary clinical settings should be carried out with caution. Moreover, a methodological limitation of our study is the absence of trial sequential analysis (TSA) to assess the robustness of our meta-analysis findings. TSA is an increasingly utilized statistical method in medical literature designed to manage type I and type II errors in meta-analyses [49,50]. It involves cumulative analysis, adjusting significance thresholds, and statistical power throughout the process.

Considering that the majority of the included studies were conducted over three decades ago, future research should involve RCTs that align with current clinical standards. Researchers should also pay careful attention to issues such as blinding and provide detailed descriptions of the randomization method to enhance the quality and reliability of study outcomes. Standardized reporting of outcomes and procedures across studies would facilitate meaningful comparisons and meta-analyses. Long-term outcomes, especially in terms of recovery trajectories, should be investigated to understand the overall impact of anesthesia choice on patient recovery. Patient preferences in anesthesia decision-making are also an issue that should be explored. Addressing these aspects in future research will contribute to a more comprehensive and clinically relevant understanding of the choice between general and regional anesthesia in hip fracture surgeries. Future meta-analyses could perform a subgroup analysis based on the years of publication to partially solve the issue of including older studies.

The comparable safety and efficacy of general and regional anesthesia observed in our meta-analysis suggest that either approach can be acceptable, depending on individual patient characteristics, preferences, and clinical contexts. Policymakers and guideline developers may find it valuable to acknowledge this flexibility and consider incorporating it into recommendations. This recognition can provide healthcare practitioners with a broader choice of options and encourage shared decision-making between patients and clinicians. Additionally, our study highlights the importance of ongoing updates to clinical guidelines to reflect contemporary evidence and advancements in anesthesia techniques.

## 5. Conclusions

Existing evidence showed that the rate of mortality in patients undergoing hip fracture surgery did not differ significantly between general anesthesia and regional anesthesia. There was no statistically significant difference between RA and GA in cardiac and cerebral complications, including myocardial infarction, cardiac failure, cerebrovascular accident, deep vein thrombosis, postoperative pulmonary embolus, renal failure, postoperative pneumonia, intraoperative hypotension, intraoperative blood loss, intraoperative blood transfusion, or duration of hospital length of stay.

## Figures and Tables

**Table 1:** Characteristics of the included studies.

First Author, Year	Country	Groups	Study Outcomes	Age (Mean ± SD)	N of Patients: Total (I/C)	Local Anesthetic
Bigler, 1985 [27]	NG	GA	Prim.—postoperative mental function and morbidity	80.1 ± 1.6	40 (20/20)	SA: 3 mL bupivacaine 0.75%
		SA		77.6 ± 2.3		
Davis, 1981 [28]	New Zealand	GA	Prim.—morbidity and mortality	81 ± 8.2	132 (64/68)	SA: tetracaine 0.5% in 6% dextrose with adrenaline 1:100,000 without barbotage in 51 patients Hyperbaric cinchocaine 0.5% in 6% dextrose in 13 patients
		SA		78 ± 8.6		
Davis, 1987 [29]	New Zealand	GA	Prim.—mortality	79.5 ± 8.8	538 (259/279)	SA: tetracaine, nupercaine or bupivacaine (optional), hyper/iso-baric
		SA				
Juelsgaard, 1998 [30]	Denmark	GA	Prim.—incidence of myocardial ischemia in atherosclerotic patients	85.7 (72–94)	43 (14/15/14)	ISA: Bupivacaine 0.5% plain SDSA: 2.5 mL bupivacaine plain
		ISA		82.2 (65–99)		
		SDSA		79.6 (72–92)		
Li, 2021 [31]	China	GA	Prim.—delirium within 7 days. Sec.—delirium characteristics, pain intensity in week 1, death at 30 days, hospital LoS, complications, and long-term and financial outcomes	77 (72–82) 77 (71–82)	942 (471/471)	SA: ropivacaine EA, NB: ropivacaine, bupivacaine, lidocaine
		SA				
		EA				
		NB				
McKenzie, 1980 [32]	UK	GA	Prim.—postoperative arterial oxygenation and intraoperative mortality	76.8 ± 1.38	100 (49/51)	SA: 1.3–1.5 mL hyperbaric cinchocaine 0.5%
		SA		74.5 ± 2.29		
McKenzie, 1984 [33]	UK	GA	Prim.—mortality at 1 year	74.2 ± 1.7	150 (75/75)	SA: 1.3–1.5 mL hyperbaric 0.5% cinchocaine
		SA		75.4 ± 1.4		
McKenzie, 1985 [34]	UK	GA	Prim.—incidence of deep vein thrombosis and pulmonary embolism	73.9 ± 4.1	40 (20/20)	SA: 1.2–1.5 mL hyperbaric conchocaine
		SA		72.3 ± 2.8		
McLaren, 1978 [35]	UK	GA	Prim.—mortality and morbidity	76 ± 9.7	55 (26/29)	SA: 0.5 mL hyperbaric cinchocaine (0.5% in 6% dextrose)
		SA		75.6 ± 10.3		
Messina, 2013 [36]	Italy	GA	Prim.—hemodynamic response	81.8 ± 6.3	20 (10/10)	SA: 7.5 mg levobupivacaine diluted from 7.5 mg/mL with 2 mL distilled water + preservative-free sufentanil 5 µg
		SA		83.9 ± 9.4		
Neuman, 2021 [37]	USA, Canada	GA	Prim—death or inability to walk independently at 60 days after randomization	77.7 ± 10.7	1572 (782/790)	Varied across study sites
		SA		78.4 ± 10.6		
Parker, 2015 [38]	UK	GA	Prim.—mortality	82.9 (range 52–105)	322 (158/164)	At the discretion of the anesthetist
		SA		83.0 (range 59–99)		
Svartling, 1986 [39]	Finland	GA	Prim.—arterial blood, pressure, arterial oxygen tension, plasma levels of cortisol	79.6 ± 2.1	30 (15/15)	SA: 3 mL isobaric bupivacaine hydrochloride 0.5%
		SA		75.1 ± 1.1		
Tzimas, 2018 [40]	Greece	GA	Prim.—POCD at 30 days after surgery, possible differences Sec.—delirium on days 1, 2, 3, 4	77.11 ± 6.5	70 (37/33)	SA: fentanyl 20 mcg + ropivacaine 0.75% based on somatometric characteristics
		SA		75.09 ± 6.08		
White, 1980 [41]	South Africa	GA	Prim.—pre, intra-, and postoperative events and mortality	78 ± 7.8	56 (20/20/16)	SA: hyperbaric cinchocaine 0.6–0.8 mL
		SA		80 ± 9.1		
		PCB		78 ± 7.3		

Abbreviations: C, control; I, intervention; N, number; POCD, postoperative cognitive dysfunction; prim., primary outcome; sec., secondary outcome; SD, standard deviation; GA, general anesthesia; SA, spinal anesthesia; ISA, incremental spinal anesthesia; SDSA, single-dose spinal anesthesia; PCB, psoas compartment block; EA, epidural block; NB, nerve blocks; NG, not given; LoS, length of stay.

**Table 2:** Cochrane risk of bias.

Study (Author, Year)	Risk of Bias Arising from the Randomization Process	Risk of Bias Due to Deviations from the Intended Interventions	Missing Outcome Data	Risk of Bias in Measurement of the Outcome	Risk of Bias in Selection of the Reported Result	Overall Risk of Bias
Davis et al., 1981 [28]	Some concerns	Low risk	Low risk	Some concerns	Low risk	Some concerns
Bigler et al., 1985 [27]	Some concerns	Low risk	Low risk	Some concerns	Low risk	Some concerns
Davis et al., 1987 [29]	Some concerns	Low risk	Low risk	Some concerns	Low risk	Some concerns
Juelsgaard et al., 1998 [30]	Some concerns	Low risk	Low risk	Some concerns	Low risk	Some concerns
Mckenzie et al., 1980 [32]	Some concerns	Low risk	Low risk	Some concerns	Low risk	Some concerns
Mckenzie et al., 1984 [33]	Some concerns	Low risk	Low risk	Some concerns	Low risk	Some concerns
Neuman et al., 2021 [37]	Low risk	Low risk	Some concerns	Some concerns	Low risk	Some concerns
Parker et al., 2015 [38]	Low risk	Some concerns	Low risk	Low risk	Low risk	Some concerns
White et al., 1980 [41]	Some concerns	Low risk	Low risk	Some concerns	Low risk	Some concerns
Messina et al., 2013 [36]	Low risk	Low risk	Low risk	Some concerns	Low risk	Some concerns
Svartling et al., 1986 [39]	Some concerns	Low risk	Low risk	Some concerns	Low risk	Some concerns
McLaren et al., 1978 [35]	Some concerns	Low risk	Low risk	Some concerns	Low risk	Some concerns
Li et al., 2022 [31]	Low risk	Low risk	Low risk	Some concerns	Low risk	Some concerns
Tzimas et al., 2018 [40]	Some concerns	Low risk	Low risk	Some concerns	Low risk	Some concerns
McKenzie et al., 1985 [34]	Some concerns	Low risk	Low risk	Some concerns	Low risk	Some concerns

**Table 3:** Summary of findings. Abbreviations: CI, confidence interval; GA, general anesthesia; GRADE, Grading of Recommendations Assessment, Development and Evaluation; N, number; RA, regional anesthesia; RCT, randomized controlled trial.

	N of Studies	Design	N of Patients		Effect	Overall Quality
Outcome			RA	GA	Relative Risk/Mean Difference [95% CI]	
Death	10	RCT	1918	1958	1.42 [0.96, 2.10]	Low ^a^ ⨁⨁⊖⊖
Intraoperative hypotension	6	RCT	564	531	1.24 [0.59, 2.60]	Low ^a^ ⨁⨁⊖⊖
Myocardial infarction	6	RCT	1759	1793	1.23 [0.54, 2.82]	Very low ^b^ ⨁⊖⊖⊖
Cardiac failure	3	RCT	1199	1233	0.85 [0.23, 3.07]	Very low ^b^ ⨁⊖⊖⊖
Cerebrovascular accident	3	RCT	492	518	0.60 [0.03, 12.83]	Very low ^b^ ⨁⊖⊖⊖
Deep vein thrombosis	3	RCT	252	242	1.36 [0.43, 4.29]	Very low ^b^ ⨁⊖⊖⊖
Postoperative pulmonary embolus	5	RCT	1294	1328	1.59 [0.61, 4.14]	Very low ^b^ ⨁⊖⊖⊖

^a^ Due to the risk of bias and inconsistency. ^b^ Due to the risk of bias, inconsistency, and imprecision. ⨁⨁⊖⊖—low quality; ⨁⊖⊖⊖—very low quality.

## Data Availability

No new data were created or analyzed in this study. Data sharing is not applicable to this article.

## Supplementary Materials

The following supporting information can be downloaded at https://www.mdpi.com/article/10.3390/jcm12247513/s1, Document S1: Search terms; Table S1: Evidence profile.
