# Supplementary material for: The Analysis of Multiple Outcomes between General and Regional Anesthesia in Hip Fracture Surgery: A Systematic Review and Meta-Analysis of Randomized Controlled Trials
Source: J Clin Med. 2023 Dec 5;12(24):7513. doi: 10.3390/jcm12247513 (PMC10743918; doi:10.3390/jcm12247513)
Supplement: Supplementary file 1 [file jcm-12-07513-s001.zip › jcm-2691000-supplementary.pdf]

Document S1. Search terms.

((((general anesthesia) AND (regional anesthesia)) OR (spinal anesthesia)) OR (epidural anesthesia)) AND (hip fracture surgery); ((("general anaesthesia" OR "anesthesia, general" OR ("anesthesia") AND "general") OR "general anesthesia" OR ("general" AND "anesthesia")) AND ("regional anaesthesia" OR "anesthesia, conduction" OR ("anesthesia" AND "conduction") OR "conduction anesthesia" OR ("regional" AND "anesthesia") OR "regional anesthesia")) OR ("spinal anaesthesia" OR "anesthesia, spinal" OR ("anesthesia" AND "spinal") OR "spinal anesthesia" OR ("spinal" AND "anesthesia")) OR ("epidural anaesthesia"OR "anesthesia, epidural" OR ("anesthesia"AND "epidural") OR "epidural anesthesia" OR ("epidural" AND "anesthesia"))) AND (("hip fractures" OR ("hip" AND "fractures") OR "hip fractures" OR ("hip″ AND "fracture") OR "hip fracture") AND ("surgery" OR "surgery" OR "surgical procedures, operative" OR ("surgical" AND "procedures" AND "operative") OR "operative surgical procedures" OR "general surgery" OR ("general" AND "surgery") OR "general surgery" OR "surgery s" OR "surgerys" OR "surgeries")).

Table S1. Evidence profile

| | Death | Intraoperative hypotension | Myocardial infarction | Cardiac failure | Cerebrovascular accident | Deep vein thrombosis | Postoperative pulmonary embolus |
|---|---|---|---|---|---|---|---|
| **Risk of bias** | **Serious** | **Serious** | **Serious** | **Serious** | **Serious** | **Serious** | **Serious** |
| Lack of allocation concealment | Unclear | Unclear | Unclear | Unclear | Unclear | Unclear | Unclear |
| Lack of blinding | Yes | Yes | Yes | Yes | Yes | Yes | Yes |
| Incomplete accounting of patients and outcome events | No | No | No | No | No | No | No |
| Selective outcome reporting | No | No | No | No | No | No | No |
| Other limitations | No | No | No | No | No | No | No |
| **Inconsistency** | **Serious** | **Serious** | **Serious** | **Serious** | **Serious** | **Serious** | **Serious** |
| $I^2$ (unexplained heterogeneity of results) | No | Yes | No | No | Yes | Yes | No |
| Wide variance of point estimates | Yes | Yes | Yes | Yes | Yes | Yes | Yes |
| Confidence intervals (CIs) do not overlap | No | No | No | No | No | No | No |
| **Indirectness** | **Not serious** | **Not serious** | **Not serious** | **Not serious** | **Not serious** | **Not serious** | **Not serious** |
| Differences in population | No | No | No | No | No | No | No |
| Differences in interventions | No | No | No | No | No | No | No |
| Differences in outcome measures | No | No | No | No | No | No | No |
| Indirect comparisons | No | No | No | No | No | No | No |

| Imprecision | Not serious | Not serious | Serious | Serious | Seriou s | Seriou s | Seriou s |
|---|---|---|---|---|---|---|---|
| Few patients | No | No | No | No | No | No | No |
| Wide confidence interval (CI) | No | No | Yes | Yes | Yes | Yes | Yes |
| **Upgrading** | **None** | **None** | **None** | **None** | **None** | **None** | **None** |
| RR>2 or RR<0.5 RR>5 or RR<0.2 | No | No | No | No | No | No | No |
| Dose-response gradient | No | No | No | No | No | No | No |
| Effect of plausible residual confounding | No | No | No | No | No | No | No |
